# The complete mitochondrial genome of *Praia tianmunica* (Hymenoptera: Cimbicidae) with related phylogenetic analysis

**DOI:** 10.1080/23802359.2020.1797576

**Published:** 2020-07-28

**Authors:** Yalan Cheng, Yuchen Yan, Meicai Wei, Gengyun Niu

**Affiliations:** a College of Life Sciences, Jiangxi Normal University, Nanchang, PR China; b Key Laboratory of Cultivation and Protection for Non-Wood Forest Trees, Ministry of Education, Central South University of Forestry and Technology, Changsha, PR China

**Keywords:** Mitogenome, next-generation sequencing, gene rearrangement, phylogeny, Cimbicidae, *Praia*

## Abstract

The complete mitochondrial genome of *Praia tianmunica* is 15,556 bp long. It contains 37 genes and a control region of 505 bp in length. Gene rearrangement is present in the mitogenome of *P. tianmunica*. The maximum-likelihood and Bayesian phylogenetic analyses showed that the genus *Praia* was closely related to genus *Labriocimbex*.

*Praia* Wankowicz, 1880 is a small Palearctic genus of Cimbicidae with four species that have been reported (Yan et al. 2019). The systematic position of the genus within Cimbicidae is uncertain at present. Deng (2000) regarded *Praia* as a sister group of (*Cimbex* + *Palaecimbex*) + *Asicimbex*. A recent cladistic analysis of Cimbicidae based on morphological characters showed that *Praia* is a basal lineage of Cimbicinae (Vilhelmsen 2019). While Yan et al. (2019) proposed *Praia* was the sister group of *Labriocimbex*, and close related to *Trichiosoma*, thus to place *Praia* into tribe Trichiosomini. In this study, we presented the complete mitochondrial genome of *P. tianmunica*, and conducted a phylogenetic analysis incorporated with other available sequences, to clarify the phylogenetic position of this genus further.

Samples of *P. tianmunica* (CSCS-Hym-MC0049) were collected in Laodian, Xitianmushan, Lin’an, Zhejiang, China (30.343°N, °119.433°E) in April 2018. Genomic DNA was prepared in 150 bp paired-end libraries, tagged, and analyzed with the high-throughput Illumina Hiseq 4000 platform. A total of 16,724,850 raw reads was obtained. DNA sequences were assembled using MitoZ (Meng et al. 2019). Further validation conducted in Geneious Prime version 2019.2.1 (https://www.geneious.com) using *Leptocimbex yaniae* as a reference (coverage = 4595). First, the intergenic region between trnH and nad4 generated by MitoZ was proved to be an error by reassembly using *nad4* as the reference in Geneious. Then, we used *trnM-trnQ* (135 bp), *trnY-trnC-trnI* (215 bp), and a randomly selected sequence from the middle of the control region (150 bp) as reference sequences to examine the longest interval region further. By consistently obtaining similar coverage of the assembly contigs, we were able to confirm the 505 bp non-coding region between *trnQ* and *trnY*. Annotations were generated in the MITOS web server (Bernt et al. 2013) and revised when necessary. The GenBank accession no. is MT665975 and the accession number of BioProject is PRJNA641767.

The complete mitogenome of *P. tianmunic*a was 15,556 bp long and contained the typical set of 37 genes and a 505 bp long control region (AT% = 86.5%). The gene arrangement of *P. tianmunica* was consistent with that of *Labriocimbex sinicus*, *Trichiosoma anthracinum*, and *T. vitellina*. Four overlapping regions and 17 intergenic regions were scattered throughout the genome.

Phylogenetic analysis was based on ten unsaturated amino acids (*atp8*, *nad2*, and *nad4L* were excluded) of 44 Symphytan species and two Apocritan species. The Bayesian inference with Phylobayes under the MtArt-CAT model (Lartillot et al. 2009) conducted on the CIPRES webserver (Figure 1). Each branch of Cimbicidae had strong support. It revealed the sister-group relationship between *P. tianmunic*a and *L. sinicus*, and then the two species together as the sister group of *Trichiosoma* + *Asitrichiosoma.* This confirmed the results based on *cox1* fragments in Yan et al. (2019). All related files in this study have been uploaded to figshare (https://figshare.com/projects/Praiatianmunica/83591).

**Figure 1.**
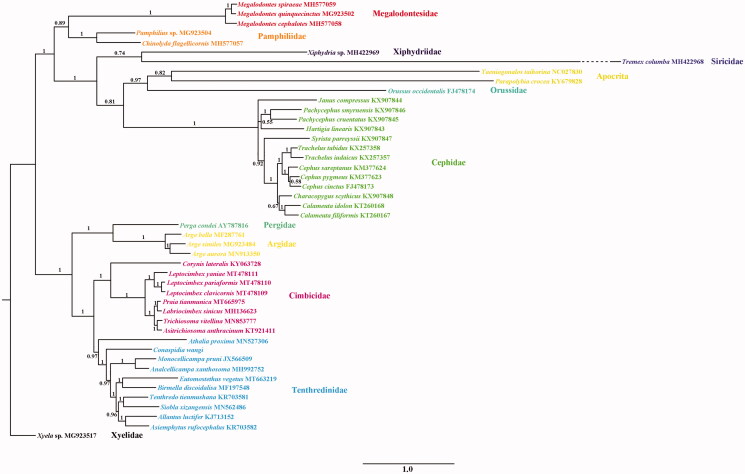
PhyloBayes tree based on the combination of ten unsaturated amino acids. Numbers on each node correspond to the posterior probability values.
